# Testing perioperative meloxicam analgesia to enhance welfare while preserving model validity in an inflammation-induced seizure model

**DOI:** 10.1038/s41598-024-81925-7

**Published:** 2024-12-19

**Authors:** Edna Weiß, Alberto Pauletti, Asya Egilmez, Sonja Bröer

**Affiliations:** Institute of Pharmacology and Toxicology, School of Veterinary Medicine, Freie Universität Berlin, Koserstraße 20, 14195 Berlin, Germany

**Keywords:** Epilepsy, Cellular neuroscience

## Abstract

Despite the international effort to improve laboratory animal welfare through the 3R principles (Reduce, Refine, Replace), many scientists still fail to implement and report their assessment of pain and well-being, likely due to concerns regarding the potential effects of analgesics on experimental outcomes. This study aimed to determine whether refining our viral encephalitis model with perioperative analgesia could enhance well-being and recovery after intracerebral virus infection without impacting disease outcomes. We routinely use the Theiler’s Murine Encephalomyelitis Virus (TMEV) model to study virus-induced epilepsy. Given the crucial role of immune cell activation in acute seizure development, we evaluated the effects of the non-steroidal anti-inflammatory drug (NSAID) meloxicam on inflammation, neurodegeneration, and neuronal cell proliferation at 7 days post-infection (dpi). Overall, the impact of virus infection on well-being was less severe than anticipated, and meloxicam treatment did not affect well-being or nest building behavior in TMEV-infected mice. Furthermore, meloxicam treatment did not influence key experimental readouts such as seizure burden, central inflammatory response, neurodegeneration, or neuronal proliferation within the hippocampus. Notably, animals experiencing seizures displayed heightened inflammatory responses and neurodegeneration, which were not influenced by meloxicam treatment. In summary, perioperative analgesia did not compromise key outcome measures such as seizure frequency, inflammation, and neurodegeneration or -regeneration in the TMEV model. However, it also did not add any significant benefits to well-being in the first week after intracranial injections.

## Introduction

More than a decade has passed since the enactment of Directive 2010/63/EU, which outlines protocols for protecting animals used in scientific research based on the principles of Replacement, Reduction, and Refinement (3Rs). These guidelines advocate for the replacement of animals with non-sentient alternatives whenever possible, the reduction of animal use, and the refinement of experimental methodologies to minimize pain and distress in experimental animals. This requires establishing minimal standards for the welfare and health of laboratory animals, including the administration of anti-inflammatory agents and analgesics in experimental procedures. However, many scientists still fail to report their approaches to pain management and refinement of well-being^1^. Neuroscience in vivo research often requires craniotomies to access the brain. A recent systematic review has confirmed that the majority of studies did not report analgesia management^2^. Insufficient attention is given to improving the conduct of animal experiments, underlining the ongoing need to implement the directive in routine animal testing practices. Experimenters may fear potential undesired effects of analgesics on their readouts, or they may require a certain level of inflammation for their model. While it is well-established that rodents can perceive pain due to the high conservation of pain signaling pathways across mammals, the tools for pain assessment remain inadequate^3^. Consequently, the under-dosing of medication due to inappropriate dosing intervals or a lack of an evidence-based dosing regimen for experimental animals remain challenges^4^. NSAIDs may be sufficient for mild to moderate pain, whereas severe pain may necessitate the use of opioids.

In this study, an NSAID was used to fulfill a request from the local animal welfare authorities for pain management during intracranial virus injections in mice. We are working with a translational model of epilepsy of infectious etiology, the TMEV model^5^. The Theiler’s virus is administered intracranially under inhalation anesthesia. While traditional research approaches in epilepsy predominantly use chemical or electrical stimulation to induce seizures, which carries a high mortality rate, the TMEV model shows advantages such as a low mortality rate, the development of chronic epilepsy after a latent period, and a high translational value^6^: About 50–75% of animals experience infection-associated acute seizures, and 20–45% develop chronic epilepsy, similar to what is observed in humans following infectious encephalitis^7–9^. Neuroinflammation can modulate epilepsy development (epileptogenesis), seizure susceptibility, and neurogenesis, making it an ineliminable component of an epilepsy model derived from an infection of the CNS^10^. Therefore, this pilot study tested whether administering an analgesic and anti-inflammatory drug to refine intracerebellar virus inoculation in an inflammation-based epilepsy model could be done without affecting disease outcome.

We selected meloxicam, a preferential cyclooxygenase-2 (COX-2) inhibitor, which provides anti-inflammatory, antipyretic, and analgesic effects. Meloxicam was administered perioperatively at a dose of 5 mg/kg to ensure adequate analgesia during intracranial virus injection. Meloxicam inhibits the synthesis of prostaglandins that promote inflammation and inflammatory pain. Inflammatory response and prostaglandin synthesis result from the activation of innate immune cells, e.g. by traumatic injury, infection, or intoxication. Meloxicam has been shown to cross the blood–brain barrier in vitro^11^ and in vivo^12^*.* The available data on its influence on seizure susceptibility remain inconclusive^13,14^. Several neurological disorders are induced or worsened by inflammation^15–17^. Recent studies have shown that seizures often accompany neuroinflammation and are likely triggered by inflammation^18^. We wanted to include this pain management in our experimental procedure without distorting the readouts in our inflammation-based seizure model. Specifically, recovery from virus infection was assessed by scoring well-being, nest building performance, and weight development in non-injected (sham) mice, vehicle-injected control (CTR) mice, and TMEV-injected mice with and without perioperative meloxicam analgesia. On the other hand, acute seizure occurrence, neuroinflammation, neurodegeneration, and cell proliferation in the brain were evaluated to determine potential interferences of pain medication with key disease outcome parameters in CTR and TMEV mice with and without meloxicam treatment^19^. Thus, the overall advantages of an analgesic drug had to be weighed against its possible effects on the model.

## Results

### Meloxicam had no influence on seizure frequency and severity after infection

**Fig. 1:**
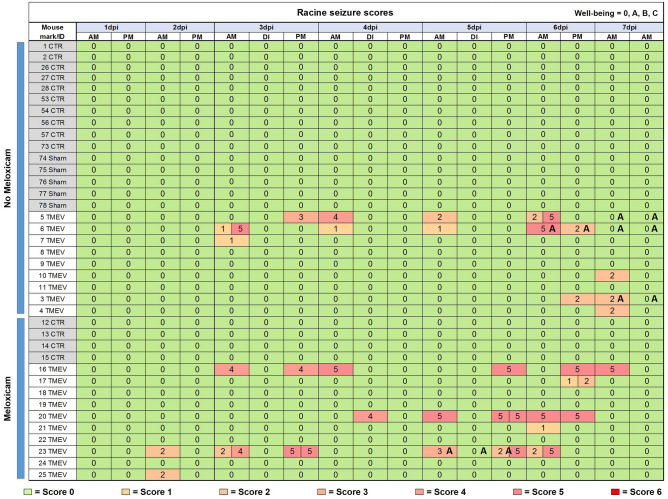
Heatmap of the Racine seizure scores and scores for well-being. Scores are shown for TMEV, vehicle or non-injected (sham) animals in the meloxicam treatment group (bottom) and the group without meloxicam (top). Behavioral seizures were observed and scored during monitoring and handling hours in the morning and afternoon for one week after infection. Twelve TMEV-infected mice showed epileptiform behavior. Seizure observation during injection (DI) of BrdU was noted likewise. Seizure stages are visualized going from green (= 0) to the highest score in red (= 6). Well-being scores (0, A, B, or C) are only shown if they were > 0. Three out of 9 TMEV-infected mice without meloxicam treatment received a score of “A” in well-being compared to only one animal in the meloxicam treatment group (n = 10). Well-being was evaluated according to a scoring sheet (see Supplementary Fig. 1).

In the days after infection TMEV animals developed an acute encephalitis, during which 12 out of 19 TMEV mice developed seizures (Fig. 1). This aligns with the reported number of 50–75% of animals developing seizures in the acute phase of a TMEV infection^19^. The seizures were scored according to a modified Racine scale ranging from 0 to 6, and are displayed in a heat-map (Fig. 1)^20^. Facial movements and head nodding (score 1 + 2) were observed in many animals, while only a few mice showed a score of 3 (myoclonus or unilateral forelimb clonus) or higher (tonic–clonic convulsions = score 4, loss of righting reflexes = score 5, excessive running and jumping = score 6). As expected, none of the CTR or sham animals showed seizure activity (score 0; Fig. 1).

**Fig. 2:**
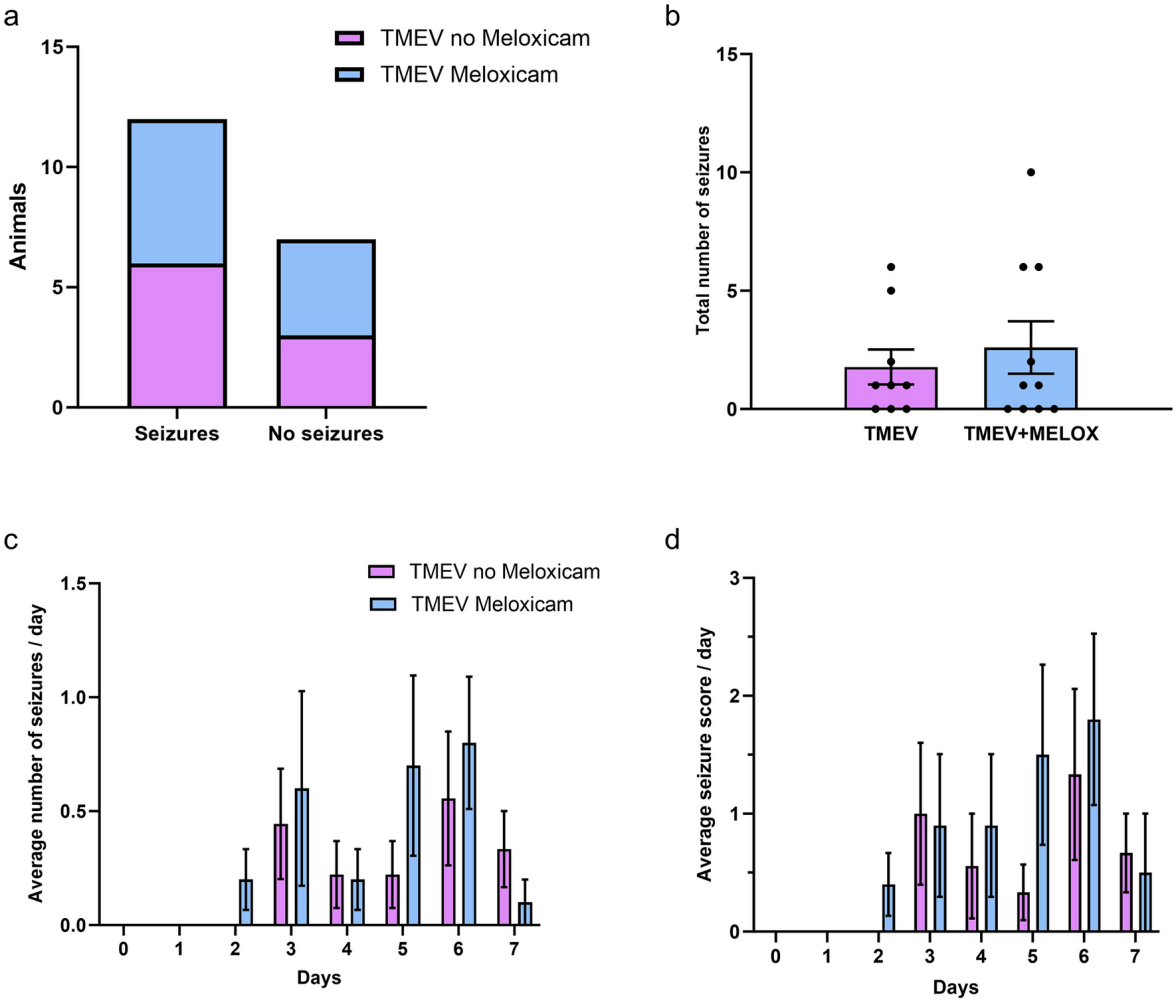
Seizure frequency and severity in TMEV-infected mice with and without meloxicam treatment. (**a**) Fisher’s exact test of meloxicam-treated and untreated mice revealed no difference in seizure incidence (P > 0.999); (**b**) Mann–Whitney test of total seizure frequency was not significant (P = 0.8593); (**c**) two-way ANOVA for average seizure frequency per day, and (**d**) average seizure severity per day was not significant. Behavioral seizures started two days post injection in TMEV meloxicam-treated mice and three days after injection in the TMEV group. Data are presented as mean ± SEM.

Some animals never showed seizures with convulsive movements during the observation time (Fig. 2a). There was no difference in seizure incidence (Fisher’s exact test): 60% of the meloxicam-treated TMEV animals developed seizures compared to 66% of the animals without perioperative analgesia (Fig. 2a). The total number of seizures was also not different between meloxicam-treated and untreated TMEV mice (Fig. 2b). We also assessed whether meloxicam application altered daily seizure frequency or severity. As reported previously in this model, most of the seizures occurred between 3 and 6 dpi (Fig. 2c)^5,8,19^. There were also no significant differences in seizure severity between animals with and without analgesic treatment. Again, the most severe seizures occurred between 3 and 6 dpi (Fig. 2d).

### Meloxicam did not have a beneficial effect on well-being after virus infection

In order to assess a potential effect of perioperative analgesia on well-being, we compared TMEV-infected animals with and without meloxicam to control animals with an intracerebral injection of vehicle (CTR) with and without meloxicam, and to a sham control group. Sham animals did not receive an intracerebellar injection and no meloxicam treatment, but underwent all other described procedures. We introduced a scoring system to analyze well-being after intracranial injection (Supplementary Fig. 1). The score ranged from 0 to C; C would have been the humane endpoint of the experiment. All animals received a score of 0 in the 24 h after virus infection, thus no differences between sham, CTR and TMEV mice with and without meloxicam could be assessed. The well-being scores were integrated into the seizure heat-map (Fig. 1). A score of A was given to four mice due to a > 10% weight loss, or reduced movement towards the end of the acute phase of encephalitis (Fig. 1). Three out of these four mice had not received perioperative analgesia with meloxicam. Interestingly, in relation to our experimental animal license, in which the burden of virus infection and following encephalitis was classified as moderate (“B”), the animals showed better than expected well-being, i.e., a lower burden.

### Meloxicam had no beneficial influence on nest building behavior

**Fig. 3:**
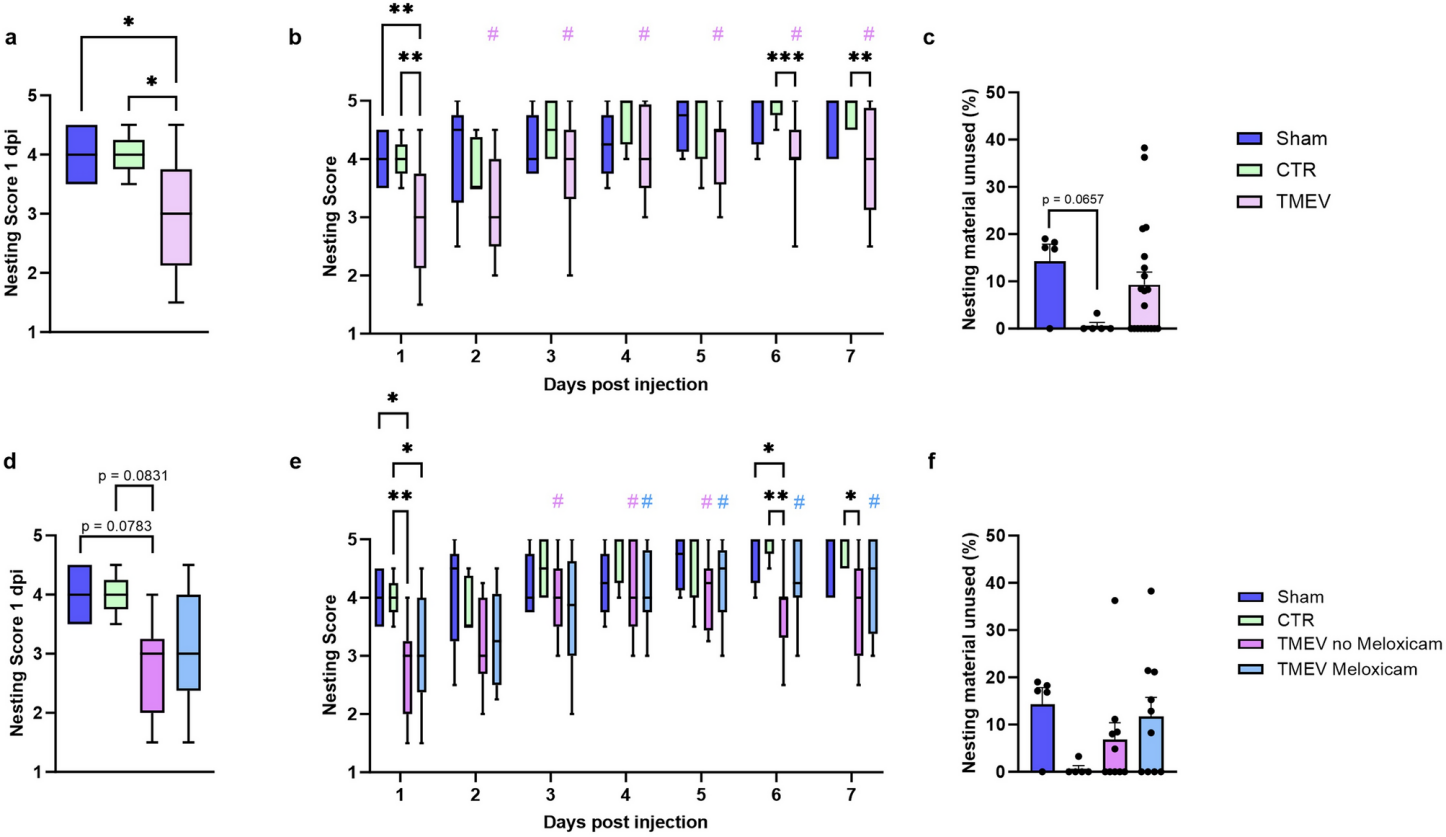
Nesting score in TMEV, CTR and sham mice with or without meloxicam treatment. Data are presented as box and whisker plots, with the bottom and the top displaying the first and third quartiles, and the band inside the box representing the median. The ends of the whiskers show the minimum and maximum of the data. Significant intergroup differences are marked as *; intragroup differences are marked as # in the respective color. (**a**) Nesting score at 1 dpi in sham (n = 5) vs. CTR (n = 5) vs. TMEV-infected (n = 20) mice. Kruskal–Wallis test followed by Dunn’s post-hoc test revealed a significantly lower score in TMEV animals compared to sham (*p = 0.0379) and CTR mice (*p = 0.0405). (**b**) Daily nesting score during the acute phase in sham (n = 5) vs. CTR (n = 5) vs. TMEV (n = 20). At 1 dpi, TMEV animals showed a significantly lower nesting score than sham (**p = 0.0088) and CTR (**p = 0.0011) animals analyzed by two way-ANOVA followed by Tukey’s post-hoc test. The difference was still present towards the end of the acute phase in CTR vs. TMEV mice on 6 dpi (***p = 0.0004) and 7 dpi (**p = 0.0057). A constant improvement in nest building performance could be seen in TMEV mice starting on day 3 (#p < 0.0001) to day 7 (#p = 0.0002) after the injection. (**c**) Remaining nesting material at 7 dpi in % in sham (n = 5) vs. CTR (n = 5) vs. TMEV mice (n = 20). No significant differences between groups were detected (Kruskal–Wallis followed by Dunn’s post-hoc test). (**d**–**f**) Influence of meloxicam treatment on nest scores: (**d**) Nesting score at 1 dpi divided into meloxicam-treated TMEV mice (n = 10) and no treatment TMEV (n = 10) did not reach statistical significance. (**e**) Daily nesting score during the acute phase in sham (n = 5) vs. CTR (n = 5) vs. TMEV meloxicam-treated (n = 10) vs. TMEV untreated mice (n = 10) by two way-ANOVA followed by Tukey’s post-hoc test. There were no differences between TMEV mice with and without meloxicam, but TMEV mice without meloxicam treatment showed lower nest scores than CTR (**p = 0.0081) and sham (p* = 0.0195) at 1 dpi, as well as at the end of the acute phase compared to sham (6 dpi *p = 0.0418) and CTR (6 dpi *p = 0.0032, 7 dpi *p = 0.0162). The TMEV meloxicam-treated mice showed a significant improvement in nest building compared to the day after injection from day 4 onwards (4 dpi #p = 0.0093, 5 dpi #p = 0.0165, 6 dpi #p = 0.0048, 7 dpi #p = 0.0019). Mice without meloxicam showed improved scores on some days (3 dpi #p = 0.0004, 4 dpi #p = 0.0174, 5 dpi #p = 0.0069). (**f**) Comparison of the remaining nesting material at 7 dpi in % in meloxicam-injected TMEV mice (n = 10) vs. TMEV mice without treatment (n = 10) was not significant.

Nest building is a reliable indicator of well-being in laboratory mice, as it stems from an evolutionary survival instinct to provide protection and safety^21^. Deviations in this behavior or nest quality are signaling changes in environmental conditions or the animals’ physiological or psychological state^22^, and can be assessed by a scoring system^23^. As the day after intracranial virus inoculation was of particular interest to evaluate potential effects of perioperatively applied meloxicam, we compared nesting-behavior for the first time at 1 dpi: TMEV-infected mice had significantly lower nest scores compared to sham and CTR mice (Fig. 3a). Nest scores significantly improved over time compared to the first day in TMEV mice, but interestingly, towards the end of the acute phase at 7 dpi, TMEV animals again presented with lower nesting scores compared to the sham and CTR group (Fig. 3b). At the end of the week untouched nesting material was weighed, and there were no differences comparing TMEV animals to sham and CTR mice (Fig. 3c). We could only detect a trend in sham animals using less material compared to CTR mice (Fig. 3c). Differences in the chosen nesting materials could be explained through different preferences since a variety of three tissues was offered.

Next we assessed differences between meloxicam-treated and untreated mice: There was no difference in nest scores between TMEV animals with and without analgesia on any of the investigated days (Fig. 3d,e), but only non-treated TMEV were different from sham and CTR groups towards the end of the experiment (Fig. 3e), while the scores in meloxicam-injected TMEV mice consistently improved from 4 dpi onwards. Animals with meloxicam treatment compared to untreated animals did not show any significant differences in the utilization of the offered nesting materials at 7 dpi (Fig. 3f).

### Meloxicam had no influence on weight development after virus infection

**Fig. 4:**
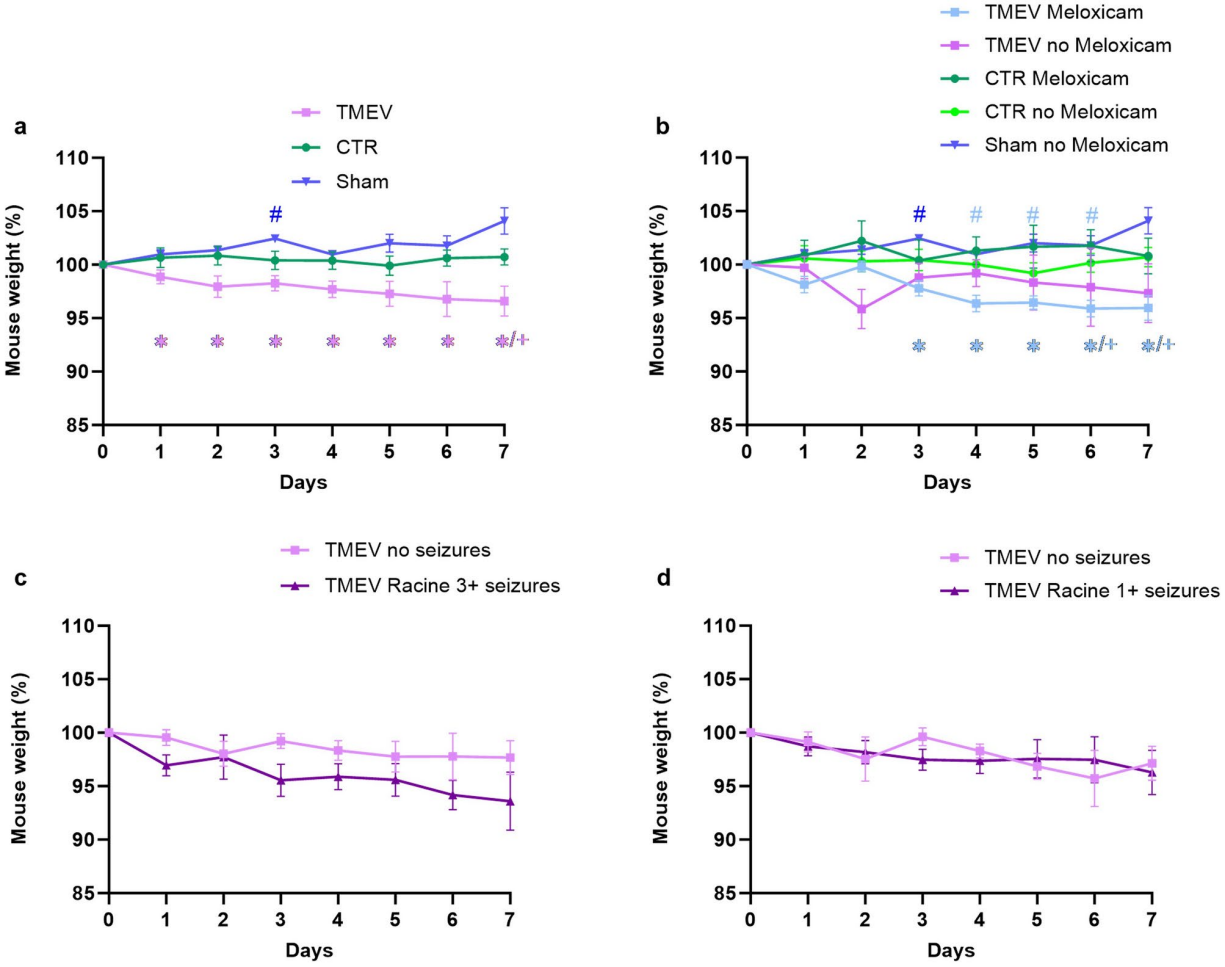
Weight development in TMEV, CTR and sham mice with or without meloxicam treatment. Weight on the day of infection was set to 100% and subsequent weights were normalized to baseline weight. Significant intergroup differences to the sham group are marked as *, intergroup differences to CTR are marked as + , and intragroup differences are marked by # in the respective group colors. (**a**) Weight development in all TMEV (n = 19) vs. CTR (n = 14) vs. sham (n = 5) was compared by mixed effects model ANOVA followed by Tukey’s multiple comparison test, and showed a significant weight loss in TMEV animals compared to the sham group on day 1 (*p = 0.0386), day 2 (*p = 0.0113), day 3 (****p =  < 0.0001), day 4 (**p = 0.0017), day 5 (*p = 0.0111) and day 6 (*p = 0.0374). On day 7 after injection, TMEV animals were significantly lower in weight than sham (**p = 0.0031) and CTR mice (+ p = 0.0397). Sham animals significantly gained weight on 3 dpi compared to their baseline weight (#p = 0.0068). (**b**) Influence of meloxicam on weight development: The body weight development of TMEV Meloxicam (n = 10), TMEV no Meloxicam (n = 9), CTR Meloxicam (n = 4), CTR no Meloxicam (n = 10), Sham (n = 5) were compared. The mixed effects model ANOVA followed by Tukey’s multiple comparison test revealed a significantly lower weight in the TMEV Meloxicam group compared to sham on day 3 (***p = 0.0005), 4 (**p = 0.0013), 5 (**p = 0.0040), 6 (**p = 0.0052) and 7 (**p = 0.0046), as well as CTR on 6 (+ p = 0.0145) and 7 dpi (+ p = 0.0337). Compared to their initial weight, TMEV Meloxicam animals lost weight on day 3 (#p = 0.0160) ,4 (##p = 0.0045) and 5 (##p = 0.0074) post-infection, which was not seen in untreated TMEV mice. (**c**) Influence of seizures on weight development: There were no differences between seizing and non-seizing TMEV mice when only Racine score 3 and above seizures were included (n = 5), and also when (**d**) all seizures were included (n = 12). Data are presented as mean ± SEM.

Another measure to assess well-being and recovery, or on the other hand to determine sickness behavior and humane endpoints in laboratory animals, is the body weight^24^. All animals were weighed before infection (day 0) and their baseline weight was set to 100%. During the first week after intracerebral injections, sham animals gained weight on day 3 compared to their baseline weight (Fig. 4a). Other than that, the body weight of sham and CTR mice did not differ compared to their own baseline weight or between the two groups on specific days. Analyzed as a whole group, TMEV infected animals had a significantly lower weight starting at 1 dpi until the end of the acute phase compared to sham injected animals. Additionally, TMEV mice displayed a lower weight compared to CTR at 7 dpi (Fig. 4a). When separating meloxicam-treated and untreated groups, there was no difference in weight development in CTR mice with and without meloxicam (Fig. 4b). A direct comparison of meloxicam-treated and untreated TMEV-infected mice also did not show any differences (Fig. 4b). However, it became apparent that only TMEV mice with meloxicam lost weight over time (4–6 dpi) compared to their own baseline weight. Their weight was also significantly lower than sham as well as CTR mice towards the end of the acute phase (Fig. 4b). Examining the influence of seizures on weight development after virus infection revealed no differences between seizing and non-seizing TMEV mice (Fig. 4c). This result was not changed by including animals without tonic–clonic seizures (Fig. 4d).

### Meloxicam did not alter neuroinflammation after virus infection

Since seizures in the TMEV model are a consequence of viral infection and encephalitis, the administration of an NSAID such as meloxicam could represent a risk for alteration of the inflammatory response and consequently the experimental outcome. For assessment of the central immune response, we examined differences in microglia and astrocyte cell count and cell area in the hippocampus as the most affected brain region in this model.

**Fig. 5:**
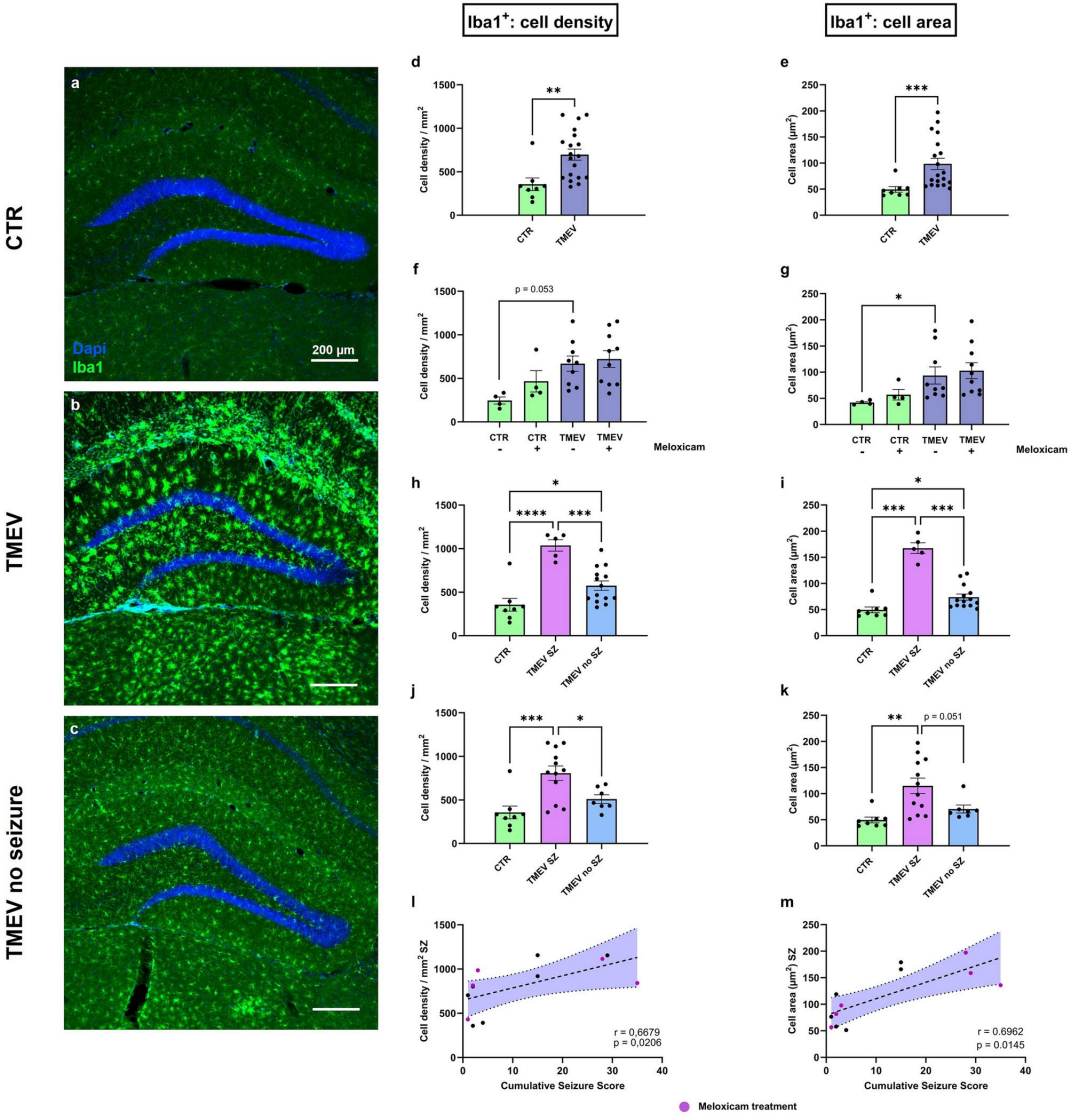
Microglia activation in CTR and TMEV-infected mice with and without meloxicam treatment. (**a**-**c**) Immunohistological labeling of microglia (Iba1^+^) in (a) CTR, (b) TMEV, and (c) TMEV no seizure animals. Highest activation of microglia with larger cell size was seen in seizing animals. (**d**) Unpaired t-test showed significantly higher cell density of Iba1-positive cells in all TMEV (n = 19) vs. CTR (n = 8) mice (p = 0.0047). (**e**) The average cell area in Iba1^+^ cells was also larger in TMEV vs. CTR animals (p = 0.0002), analyzed by Mann–Whitney-test. (**f** + **g**) Influence of meloxicam treatment on microglia activation: Analysis of cell density/mm^2^ and average cell area in CTR with (n = 4) and without meloxicam (n = 4), and TMEV mice with (n = 10) and without meloxicam (n = 9). There were no differences detected between meloxicam-treated and untreated groups by Welch ANOVA test followed by Dunnett‘s multiple comparison test. (**h**) Influence of seizures on microglia activation: Differences between CTR (n = 8), TMEV with Racine score 3 or higher seizures (n = 5) and without seizures (n = 14) were analyzed by one-way ANOVA followed by Tukey‘s multiple comparison test. Seizing animals had significantly higher cell density than CTR (p < 0.0001) and TMEV mice without seizures (p = 0.0004). Seizure free animals still had a noteworthy higher cell density compared to CTR (p = 0.0459). (**i**) Mean cell area was significantly higher in seizing animals with a Racine score 3 or higher compared to CTR animals (p = 0.0001) and TMEV without seizures (p = 0.0003) in a Welch ANOVA test followed by Dunnett‘s multiple comparison test. Seizure free TMEV mice had a significantly larger cell area compared to the CTR (p = 0.0198). (**j** + **k**) Analyses including all animals with seizure behavior (n = 12). Mean cell density (p = 0.0008) tested with ANOVA and mean cell area (p = 0.0031) analyzed with Welch ANOVA were significantly higher in seizing mice vs. CTR. Cell density was significantly higher in seizing than in non-seizing animals (n = 7) (p = 0.0346). (**l** + **m**) Correlation between the cumulative seizure score and cell density (p = 0.0206) or cell area (p = 0.0145) was significant. Data are presented as mean ± SEM.

Immunohistological analyses of Iba1 marked cells in the dentate gyrus (DG) revealed a higher cell number and intensified fluorescence signal in TMEV animals (Fig. 5a–c). As a quantitative confirmation, investigation of overall differences in averaged Iba1^+^ cell count per mm^2^ after virus infection (Fig. 5d) or averaged cell area (Fig. 5e) showed a significantly higher value in the TMEV-infected animals compared to CTR mice. The higher cell number and increase in cell size indicate the response of microglia to an inflammatory event and is consistent with data from the literature^18^. We then investigated the effect of meloxicam treatment: We could not detect any significant differences in microglial activation between animals with and without analgesia in CTR or TMEV mice (Fig. 5f + g). Consequently, we conclude that the perioperative injection of meloxicam during virus infection had no effect on the inflammatory response of microglia 7 dpi in the TMEV model.

Furthermore, we wanted to evaluate whether seizures in TMEV mice had an influence on microglia activation (Fig. 5h–m). TMEV infected mice with and without seizures showed more pronounced microglial activation compared to CTR (Fig. 5h + i), with convulsive seizing mice (Racine 3 or higher) displaying the highest microglial activation (Fig. 5h + i). Pronounced activation of microglia was visible especially in the seizing animals, noticeable by retracted protrusions and a higher number of cells (Fig. 5b). Data including non-convulsive seizure activity (Racine 1/2) (Fig. 5j + k) was consistent in showing a significantly higher cell density and cell area in TMEV seizure animals compared to CTR. Additionally, we could detect a higher average cell density in seizing versus non-seizing infected animals. Nevertheless, we could not identify differences between the CTR group and animals without seizures, indicating a correlation between seizures and inflammation. In fact, the cumulative seizure score significantly correlated with the average Iba1^+^ cell density (Fig. 5l), and area (Fig. 5m). The cumulative seizure score included all electroconvulsive behavior (Racine score 1 to 6). Animals belonging to the meloxicam treatment group were equally scattered within the linear regression model, supporting its lack of influence on neuroinflammation in this model.

**Fig. 6:**
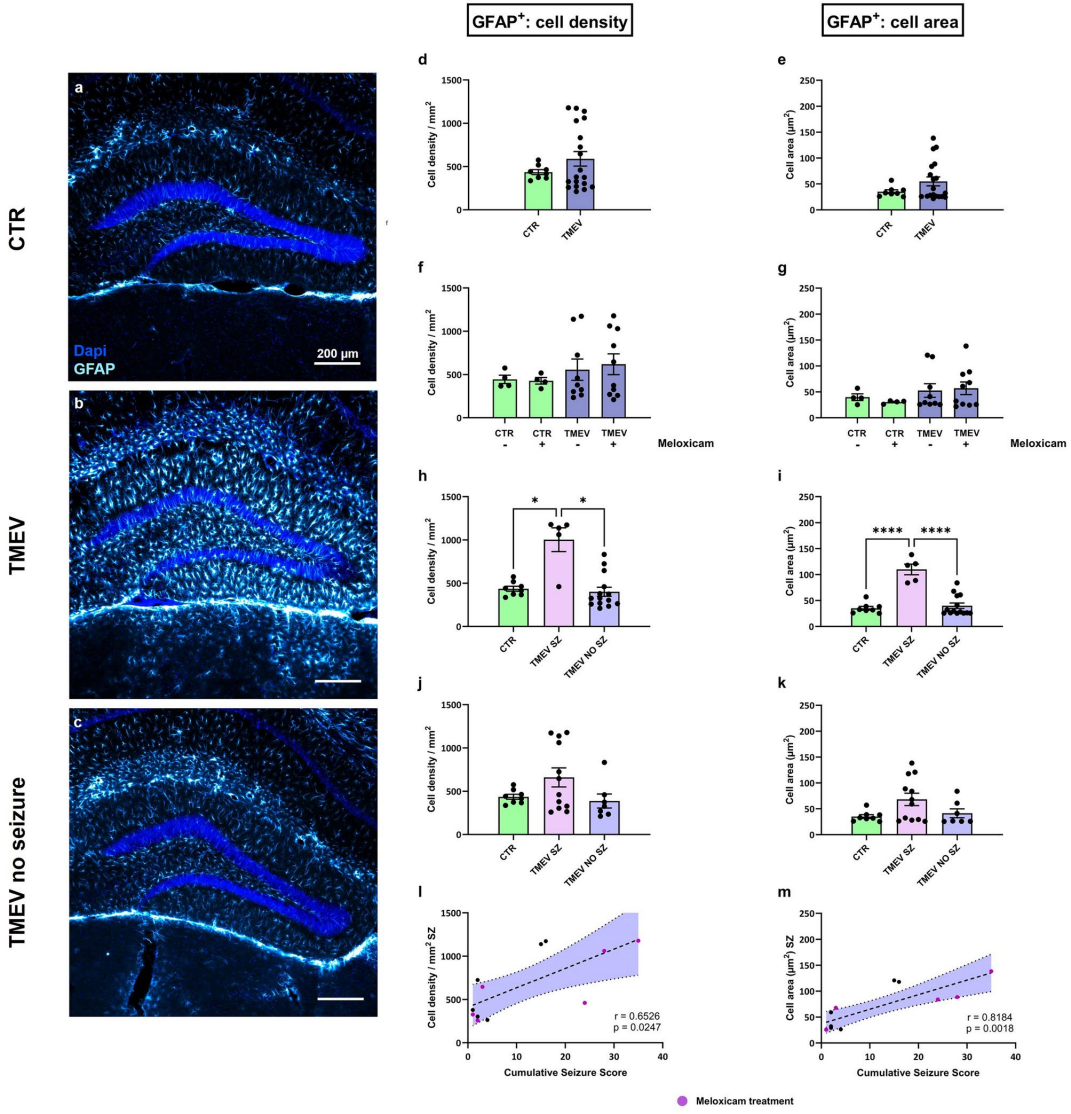
Astrocyte activation in CTR and TMEV-infected mice with and without meloxicam treatment. (**a**-**c**) Immunohistological labeling of astrocytes (GFAP^+^) in (**a**) CTR, (**b**) TMEV, and (**c**) TMEV no seizure animals. Highest activation of astrocytes with larger cell size was seen in seizing animals. (**d** + **e**) No significant differences were detected in mean cell density per mm^2^ and mean cell area comparing GFAP-positive cells in all TMEV (n = 19) vs CTR (n = 8) mice. (**f** + **g**) Meloxicam treatment had no influence on astrocyte density or size (Welch ANOVA followed by Dunnett‘s multiple comparison test for cell density/mm^2^ and Kruskal–Wallis ANOVA with Dunn‘s multiple comparisons test for cell area). (**h**) Seizing animals with Racine score 3 or higher seizures (n = 5) had significantly higher cell density than CTR (n = 8; p = 0.0391) and TMEV mice without seizures (n = 14; p = 0.0247). (**i**) Mean cell area was also significantly higher in seizing animals with a score 3 or higher compared to CTR (p < 0.0001) and TMEV without seizures (p < 0.0001), one-way ANOVA test followed by Tukey‘s multiple comparison test. (**j** + **k**) Analyses including all animals with seizure behavior (n = 12) showed no significant differences in cell density and cell area. (**l** + **m**) Correlation between the cumulative seizure score and GFAP cell density (p = 0.0247) or cell area (p = 0.0018) was significant. Data are presented as mean ± SEM.

Astrocytes in infected mice showed a similar state of activation: In comparison to the CTR group a striking increase in GFAP-positive cells could be seen in the hippocampus of seizing TMEV animals (Fig. 6a–c). However, when all TMEV mice were compared to CTR, they did not display any significant differences in the average cell density per mm^2^ nor the average cell area (Fig. 6d + e). Astrocyte number and activation did not change after meloxicam treatment (Fig. 6f + g). As a conclusion, the use of a single dose of meloxicam perioperatively during virus infection had no influence on the astrocytic immune response in the acute phase after infection. However, TMEV mice with seizures had a significantly higher mean cell density per mm^2^ compared to non-seizing TMEV and CTR mice, whereas no deviation between CTR and TMEV mice without seizures were seen. Regarding cell morphology changes, in TMEV seizure mice, GFAP-positive cells were significantly larger than in the CTR group and TMEV mice without seizures (Fig. 6h + i). This effect was lost if animals with mild Racine scores 1 and 2 seizure activity were included in the analysis (Fig. 6j + k). Possibly convulsive seizure activity and immune cell activation are linked. Evidence for this is bolstered by the significant positive correlation between the cumulative seizure score and the average cell density or the mean cell area of GFAP (Fig. 6l + m).

### Meloxicam did not prevent substantial neuronal loss nor altered cell proliferation in the hippocampus after infection

**Fig. 7:**
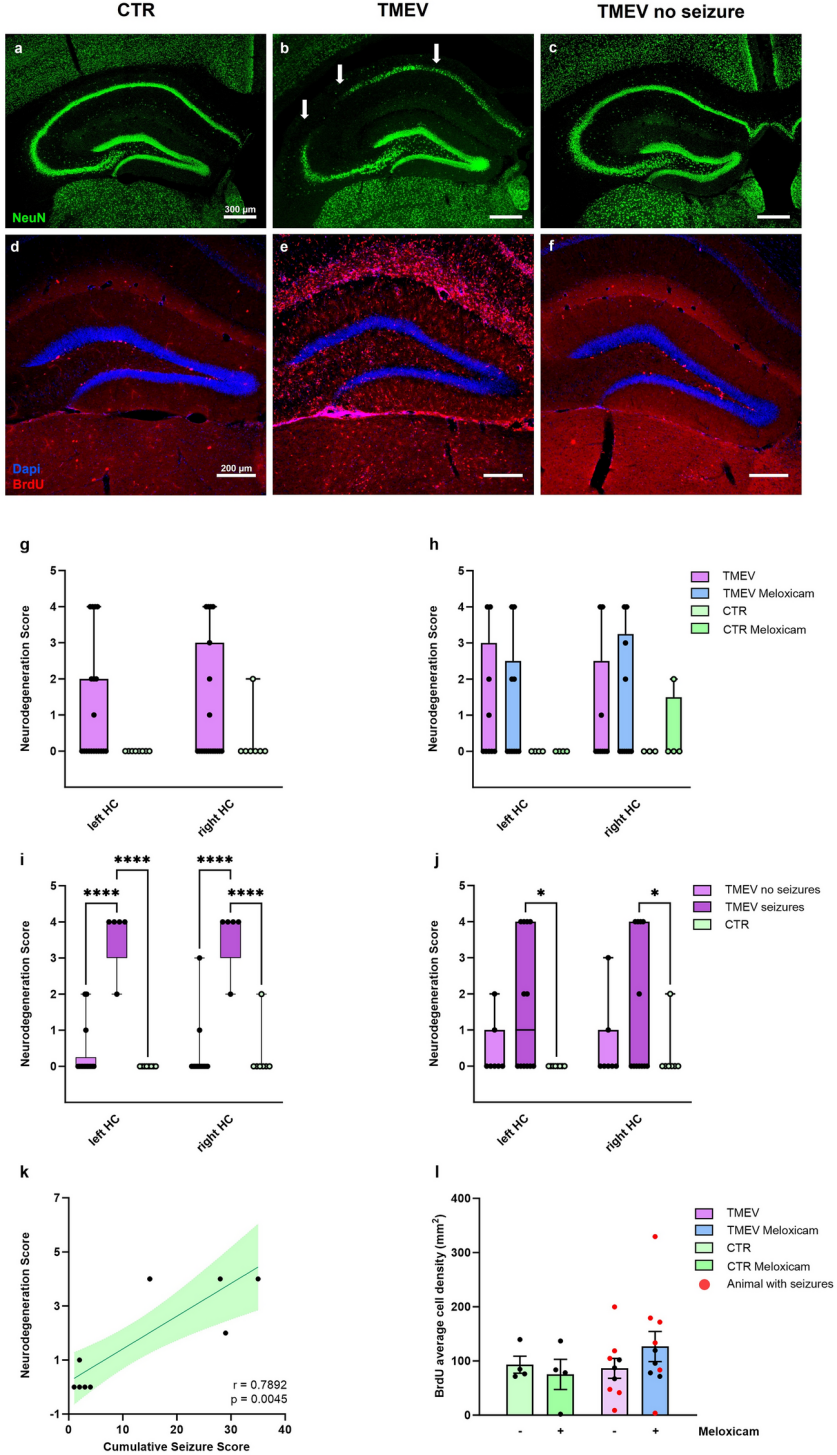
Neurodegeneration and neurogenesis in CTR and TMEV-infected mice with and without meloxicam treatment. (**a**-**c**) Immunohistochemical labeling of neurons (NeuN^+^) with apparent high cell loss in (**b**) TMEV seizure animals (white arrows). (**d**-**f**) Neurogenesis: Number of proliferating cells (BrdU^+^) within the dentate hilus is highest in (**e**) seizing animals. (**g**-**j**) Neurodegeneration was scored at 7 dpi and is plotted as box and whisker plots displaying the median, and the first and third quartile. The ends of the whiskers present the minimum and maximum. Data is divided in left and right hemisphere to check for differences after unilateral virus injection. (**g**) Differences in neurodegeneration score in all TMEV animals vs. CTR did not reach statistical significance. (**h**) Influence of meloxicam treatment on neurodegeneration: Two-way ANOVA analysis with Šidák‘s multiple comparison test revealed no differences between animals with and without meloxicam treatment. (**i**) Influence of seizures on neurodegeneration: Animals with Racine seizure score 3 or higher showed a significantly higher neurodegeneration score in both hemispheres compared to CTR (left p < 0.0001; right p < 0.0001), and TMEV mice without seizures (left p < 0.0001; right p < 0.0001). (**j**) Including Racine seizure score 1 and 2 mice, seizing mice displayed a significantly higher loss of neurons compared to CTR in the left (p = 0.0241), and right (p = 0.0241) hemisphere (two-way ANOVA followed by Šidák‘s multiple comparison test). No differences between left and right hemisphere were detected. (**k**) Correlation between cumulative seizure score and neurodegeneration was highly significant (p = 0.0045). (**l**) No significant differences in cell proliferation (BrdU^+^) between meloxicam-treated and untreated CTR and TMEV animals could be detected. Neurogenesis data is shown as mean ± SEM. For animal numbers, see Fig. 5.

We wanted to assess if the use of meloxicam for pain management during the virus infection could alter the model inherent neuronal cell loss (NeuN^+^ labeled cells) or proliferation (5-Brom-2′-deoxyuridin^+^ (BrdU) labeled cells). In CTR animals, NeuN-positive neurons along the Cornu Ammonis (CA) areas and DG were visible without interruption (Fig. 7a). Animals that received a Racine score > 3 in the seizure group showed neuronal cell loss of 20–50% or more mainly in the CA areas, most frequently in the CA1. In some areas, a complete discontinuation of the stratum pyramidale was present (Fig. 7b). In TMEV animals without seizures, the hippocampal neurons were largely undamaged, with a few animals showing a partial neuronal loss in CA1 and CA2 (Fig. 7c). When all TMEV-infected mice were analyzed as a whole, neurodegeneration was not significantly different to CTRs. The virus was injected into the right hemisphere, but there were also no differences in neurodegeneration between the left and right hemispheres (Fig. 7g). Regarding the influence of meloxicam on neurodegeneration, we examined no significant differences between TMEV animals with and without treatment (Fig. 7h). Therefore, a single application of a COX-2 inhibiting NSAID during model induction did not protect from neuronal cell loss. Given that TMEV animals did not differ from CTR mice, we assessed if seizures altered neurodegeneration. Neurodegeneration scores in the CA areas in seizure animals were significantly higher compared to CTR and seizure-free TMEV mice (Fig. 7i). When Racine stage 1 and 2 seizures were included, the difference was only visible when comparing seizing animals to CTR (Fig. 7j). Explorative correlation analyses between seizure severity expressed as cumulative seizure score and the neurodegeneration score was highly significant (Fig. 7k). Thus, the more frequent and severe the seizures were, the more neuronal cell loss was observed in the CA regions of the hippocampus. Scores did not differ comparing the left and right hemisphere.

In current projects, we are also investigating neuronal repair and cell proliferation in the TMEV model. Proliferation of new stem cells in the brain is an important readout for the understanding of cell maturation and migration after seizure insult. Since it was possible that an anti-inflammatory treatment at the time of model induction could alter the proliferative state of the neural stem cell niche in the dentate hilus, we examined TMEV-injected and CTR animals for changes in the number of proliferating cells. BrdU was injected during the acute encephalitis to label these cells. Histological analyses showed a higher number of BrdU-positive cells in seizure animals (Fig. 7e), whereas CTR and non-seizing TMEV animals only presented with a few BrdU-positive cells in the hilus of the DG, one of two areas in which mature neurogenesis occurs (Fig. 7d + f). Statistical analyses did not reveal differences in the number of BrdU^+^ cells in the dentate hilus between CTR and TMEV-injected mice. There were also no differences in comparing the number of BrdU^+^ cells between meloxicam-treated and untreated mice in either group (Fig. 7l). Mice with and without seizures were distributed evenly across the range of BrdU^+^ cells.

## Discussion

In this study, we demonstrated that a single injection of meloxicam at a dose of 5 mg/kg can be used perioperatively as an analgesic during intracranial injections without negatively affecting key experimental outcomes, including seizures, neuroinflammation, and neurodegeneration in a model of encephalitis-induced epilepsy. While intracranial injection of vehicle did not lead to any changes in well-being compared to non-injected sham mice, virus injections did lead to reduced well-being, lower nest building performance, and weight loss during the first week after infection. However, perioperatively injected meloxicam did not provide any beneficial effects on measures of well-being or an accelerated recovery after viral infection. It is noteworthy to mention that the overall impact of the intracerebral virus infection procedures, and the presumably associated pain, were generally lower than expected.

The brain itself is devoid of nociceptors, and therefore does not directly experience pain. However, pain can be generated by nociceptors located in surrounding structures such as the meninges, blood vessels, and the periosteum, which is the dense connective tissue enveloping bones^25^. Therefore, one could conclude that intracerebral injections can be perceived as painful in mice. Even if the perception of post-operative pain in craniotomies is still the subject of controversial debate and only a minimal perception of pain has long been assumed, recent data derived from human patients suggest otherwise^26^: The physical stimulation from incision and traction during a craniotomy was shown to stimulate specific nociceptors, leading to postoperative pain^27^. Thus, in craniotomies, such as stereotaxic surgeries, multimodal analgesia with added local anesthesia might be necessary. However, analysis of various pain and distress parameters following neurosurgical procedures in mice did not demonstrate a clear benefit of multimodal analgesic regimens over high-dose NSAID monotherapy^28^. This indicates that, while multimodal analgesia is widely recommended, it may not consistently offer superior outcomes. Meloxicam is the most commonly used drug for craniotomies^2^. Although a variety of doses for meloxicam have been reported, the most frequently used dose for analgesia is 5 mg/kg in experimental mice^29,30^. It is also the highest recommended dose by the National Society of Laboratory Animal Science to ensure a perioperative analgesic effect^31^. Lower doses could not preserve therapeutic plasma levels in a once-daily subcutaneous (s.c.) injection^32^. On the contrary, a literature review by Foley and colleagues (2019) identified common doses of 1 to 5 mg/kg meloxicam by oral or peritoneal route at a dosing interval of 12 h to be suitable^4^. Further, Roughan (2016) found an anti-inflammatory effect of a single s.c. injection of meloxicam only at higher doses after laparotomy in mice, but no effect of meloxicam on pain-associated parameters^33^. Contrary to that, a study by Kim and colleagues (2023) could show an increased morbidity and mortality in CD1 mice treated with a 20 mg/kg loading dose while comparing different doses of meloxicam for mouse laparotomy^34^. We did not observe any adverse effects of meloxicam injection despite a weight loss in TMEV mice with meloxicam treatment several days after treatment. It has been reported that prolonged analgesia, specifically with opioids, can induce weight loss^2^, however it is unlikely that a single NSAID treatment could lead to such a delayed effect. Instead, the weight loss coincided with the onset of encephalitis and acute seizure activity. Furthermore, meloxicam-treated CTR mice did not show any weight loss during the experiment.

Our research methodology was designed for a single injection of meloxicam perioperatively as a monotherapeutic approach. Therefore, the therapeutic effect of analgesia was limited to the time of virus infection and postoperatively associated pain. With comparable concentration–time profiles of different injection routes, the biphasic elimination of meloxicam occurs between 1–8 h after dosing with 10 mg/kg^35^. Encephalitis arises in the days after infection, and is thus not covered by the therapeutic duration of action of meloxicam. Therefore, we do not believe that the slightly higher impact on the well-being scores in non-meloxicam-treated TMEV mice on day 5–7 after infection can be attributed to not using meloxicam perioperatively. Our results suggest that a single meloxicam treatment did not exert an influence on recovery from infection, well-being, nest score, and body weight development over the acute phase of encephalitis. One could argue that administration throughout the acute phase of encephalitis would lead to a difference in experimental outcome and well-being**.** However, the peak of neuroinflammation in this model is reached around 3–5 dpi, and is a driving factor for the development of seizures^19,36^. It has been shown that the level of inflammation directly correlates and predicts seizure development in various epilepsy models^18^, including the TMEV model. Our results on microglia and astrocyte activation confirm this data. Therefore, inflammation should not be altered and prolonged anti-inflammatory treatment is not feasible for epilepsy models. On the other hand, the analgetic effect of meloxicam was desired. The virus was administered through intracranial injection through the periosteum and dura mater with a 30G needle. While this could possibly be leading to a mild perception of pain, the advantage in the chosen method is that surrounding structures remain intact, because no incision or bone burr holes have to be made. The whole procedure takes about 20–30 s under general isoflurane anesthesia. Nociception and the potential analgesic effect of meloxicam could not be assessed during the very short-term anesthesia; thus, we cannot rule out benefits of analgesia during the intracranial injections, and would advise researchers to perform additional analgesia during anesthesia whenever possible. However, the intracerebral injection itself did not produce any significant changes in well-being scores, nest building, or body weight development in the week after injection, as evidenced by comparisons between non-injected sham and vehicle-injected CTR mice.

A limitation of this study is the inherent subjectivity of a scoring system for stress and burden on the experimental animals. Without objective and validated measures of postoperative pain, current recommendations regarding choice of analgesic drug and dose remain vague. While numerous studies suggest the Mouse Grimace Scale (MGS) as an efficient parameter for pain detection in rodents post-surgery^37,38^ there is a lack of information on the feasibility in different mouse strains, age levels and surgical procedures^39^. Additionally, isoflurane anesthesia appears to distort the MGS in some mouse species^40^. In testing the reliability of the MGS in different scenarios, Hohlbaum et al. (2020) demonstrated potential difficulties for consistent assessment especially in more inexperienced observers^41^. In contrast, well-designed scores for the assessment of post-surgical pain and well-being seem to be equally suitable for pain assessment depending on the model^22,42^. These findings raise important questions regarding the feasibility of available pain assessment in rodents, as weaknesses seem to be present in each of the approaches, even if combined. A possible solution could be the implementation of artificial intelligence tools for pain assessment. Based on our observations, the assessment of pain with a scoring system was suitable for this study purpose, as evaluated parameters were appropriate for a wide range of pain-related changes in the mouse, such as movement, food intake and weight, exterior appearance, and behavior. Furthermore, similar scoring systems have been used previously in this model^19^. We decided against analysis of the MGS as a further monitoring measure, as seizure-related symptoms such as freezing, hyperexcitability or tonic–clonic seizures make evaluation more difficult. We have added widely accepted parameters for severity assessment by including nesting behavior and body weight measurements^28^ to complete our assessment of well-being.

A factor that we have not addressed is the influence of sex on efficiency of analgesia. It is well accepted today that females are more sensitive to pain, which is likely caused by gonadal hormone changes and differences in immune mediated activation of endogenous opioids^43,44^. Future studies should therefore investigate the difference in analgesia at the same dose in male and female mice. In this study, only male mice were used for assessment of the influence of meloxicam on experimental readouts, as follow up experiments are planned to be compared to other epilepsy models, in which mostly male animals are used because they experience more consistent seizure types and frequencies^45^. In the TMEV model seizure frequency is not influenced by sex^8^.

We have demonstrated that meloxicam also had no influence on the acute phase of encephalitis in the TMEV model: Seizure rates, inflammation, neurodegeneration, and -proliferation were not altered by meloxicam treatment, and showed comparable results to previously published studies^8,19^. It is known that inflammation plays an indispensable role in epileptogenesis^18,46^, specifically in this model^19,47,48^. Although the testing was required and limited to meloxicam by the authorities, it may apply also to other oxicams. Metcalf et al. investigated the efficacy of prototype anti-seizure medications (ASMs) and anti-inflammatory compounds on 3–7 dpi with once or twice-daily drug injection in the TMEV model: While several ASMs reduced seizure burden as expected, it was only moderately reduced by anti-inflammatory drugs such as celecoxib, dexamethasone, and prednisolone, but not ibuprofen and diclofenac^49^. Despite its ability to cross the blood–brain barrier, a single dose of meloxicam did not alter the central immune response in this study. A reason could be the delayed peak in immune cell activation between 3–5 dpi^47^. Although inflammation is reported to have an impact on neurodegeneration^50^, we could not detect a neuroprotective effect following meloxicam treatment, which also supports an unaltered immune response. The integrity of the blood–brain barrier is disturbed by anesthesia, infection, and encephalitis, allowing also peripheral immune cells to enter the CNS and exacerbate neuroinflammation^47,48,51^. As a consequence, peripheral activation and recruitment of immune cells have pro-epileptogenic effects. We did not assess the effect of meloxicam on peripheral immune cell activation in this study.

**Fig. 8:**
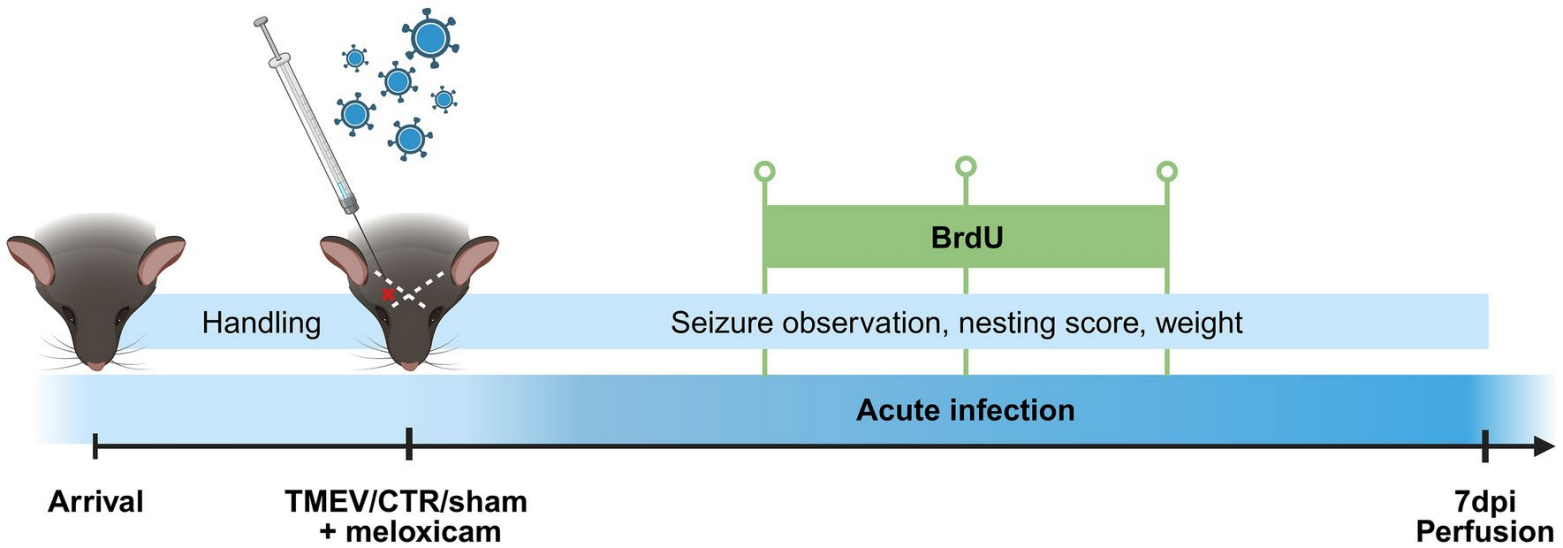
Schematic of the experimental paradigm timeline. Animals arrived on day 0. After an acclimatization period of two weeks, mice were infected with TMEV in the right parietal cortex. Meloxicam was administered perioperatively. Nest building was evaluated once daily in the morning during the acute phase of virus infection. Seizures started around day 3 after injection. As an in vivo marker for proliferating cells, BrdU was injected intraperitoneally on three consecutive days. Until perfusion at 7 dpi, mice were observed for well-being and seizures at least twice daily. This image was created with BioRender.com.

In addition to the proper use of analgesics and anesthesia, there are numerous other possibilities to refine animal experiments. Some procedures are easy to implement in daily animal handling and include enrichment of home cages, handling with tunnels or cupping as a pick-up method^52,53^. As stress is a well-recognized source of variability in experimental readouts, the comfort and health of laboratory animals can no longer take second place. Additionally, bioinformatic approaches to evidence-based comparative severity assessment might be suitable, taking into consideration the multidimensional character of distress^54^. Application of a computational workflow would allow an individualized severity classification and the selection of suitable selected behavioral and biochemical parameters in different mouse strains and models. One key point for refinement of animal experiments remains the implementation of guidelines, such as ARRIVE, in scientific reporting. In order to enhance research reproducibility detailed reporting of the methods should be observed more conscientiously. This could also reduce unnecessary experimental replica and therefore contribute to the 3Rs.

## Conclusion

Despite the critical importance of animal welfare in lab experiments, scientists often neglect to detail their analgesia and surgical care methods in peer-reviewed journals. Monitoring of well-being is still underrepresented in publications; we should move towards reporting it more accurately to close the gap between welfare directives and real-life laboratory work. In this study we showed that while refining our inflammation-dependent seizure model by adding perioperative analgesia during intracerebral injections did not offer benefits to animal welfare in the days after the procedure, it also did not negatively impact key experimental outcomes of the model. This might also be an inclination to re-evaluate the stress level and burden of procedures, as well as the need for analgesia in experimental animal models.

## Materials and methods

### Animals

In this study, we used 58 male C57BL/6J (B6) mice aged five to six weeks, purchased from Charles River Laboratories (Sulzfeld, Germany). B6 mice were chosen, as it could be shown that this particular strain was most susceptible for developing infection-induced seizures^8,19^. There is no difference in acute seizure frequency in male and female mice^8^; however, we decided to use male mice because there might be an influence of the menstrual cycle on the timing of seizure occurrence that we wanted to rule out^55^. Furthermore, the study was conducted in comparison to other epilepsy models using only male animals.

After arrival, groups of 4–5 animals were randomized into Macrolon cages Type 3 (37.5 cm × 21.5 cm × 15 cm) with nesting material as well as metal and cardboard tunnels for enrichment. The mice were housed in the animal facility of the Institute of Pharmacology and Toxicology at Freie Universität Berlin under standardized environmental conditions (light–dark cycle of 12 h, with a light phase from 6–18 h CET, at a constant temperature (21 ± 0.5 °C) and air humidity (50 ± 1%). The mice were fed a standard rodent diet (V1534, sniff Spezialdiäten GmbH, Soest, Germany) and had access to tap water and food ad libitum. The animals were given at least an acclimatization time of one week. We started to handle the mice a few days before the first procedures for letting them get used to experimenters, the surroundings, and general procedures such as lifting, weighing or fixation (Fig. 8). Assignment to study groups was conducted by simple randomization through the rand function in MS Excel: CTR animals with analgesic treatment (n = 4), CTR animals without analgesic treatment (n = 9), TMEV animals with analgesic treatment (n = 20), TMEV animals without analgesic treatment (n = 19), and to a sham control group (n = 5). Sham animals did not receive an intracerebellar injection, but underwent all other described procedures.>>>Fig .8 should be inserted HERE

### Ethics

Procedures involving animals and their care were conducted in conformity with the institutional guidelines and in compliance with national and international laws and policies (EEC Council Directive 2019/1010, June 5, 2019; Directive 2010/63/EU for the protection of animals used for scientific purposes). Our protocol was approved by the local government (LAGeSo Berlin, Germany, permission number G0015/21). We followed the recommendations of the ARRIVE guidelines. Humane endpoints in group-housed mice were based on a scoring system for well-being and pain (see Supplementary Fig. 1).

All experiments were designed and planned to minimize the number of animals used. Experimental sample size calculation was performed using G Power version 3.1 (University Düsseldorf) in consultation with the Institute of Veterinary Epidemiology and Biometrics of the Freie Universität Berlin.

### TMEV infection and seizure monitoring

#### Seizure model

The TMEV model for temporal lobe epilepsy is a translational model of infection-triggered acute and chronic seizures in the mouse, which was first described in 2008^8^. It mimics the development of acute seizures and chronic epilepsy as a consequence of viral CNS infections in humans. Our group has extensively characterized the role of immune cell activation, inflammation, and neuronal degeneration on the occurrence of seizures in this model^19,47,56,57^. The Theilervirus is a member of the family of picornaviridae, and is a single-stranded RNA mouse cardio virus. There a several different strains which can be differentiated by their virulence and pathology. We were using the Daniel’s Strain (DA) of TMEV because it reliably causes seizures, and is not as virulent as others, resulting in a low mortality risk. Depending on the mouse strain, virus strain, and viral load, about 50–75% of infected B6 mice experience acute seizures in the first 7 dpi. The virus is cleared after approximately 14 days and expectedly one third of a cohort is developing chronic epilepsy with spontaneous seizures afterwards^9,19^.

Administration of the virus was performed through non-stereotactical, intracortical injection into the right hemisphere of the mouse brain, as described in detail previously^5^. The exact injection point was found by drawing two imaginary crossing lines between the ear and the eye of the animal, then going 2 mm to the right side of the temporal lobe from the crossing in a depth of 2.5 mm (Fig. 8). The virus was diluted in Dulbecco’s Modified Eagle Medium (DMEM) and stored in a − 80 °C freezer until use. Shortly before the injection, the virus was thawed, kept on ice and drawn up into sterile syringes after resuspension. For the procedure, the mice were deeply anesthetized with isoflurane and checked for anesthesia depth via toe pinching and breathing observation. After assuring a tolerable anesthesia state, the injection site hair and surface were disinfected with isopropyl medical swabs. To assure safer handling, the skin was tightened to the neck and the head tilted slightly to the left for perpendicular injection. TMEV animals were injected with a dose of 20 µl virus suspension at a titer of 4.8 × 10^8^, and CTR mice with 20 µl of medium only, both using a 30 G insulin syringe. Sham mice underwent anesthesia, and disinfection, but no intracerebral injections. To prevent leakage, the needle was kept in place for 10 to 15 s after injection. Eye ointment was added after the injection to prevent drying of the cornea during anesthesia. Animals were then observed closely in a clean cage on a heating pad until recovery.

#### Meloxicam injection

Briefly, meloxicam was injected 15 min prior to anesthesia induction at a dose of 5 mg/kg s.c. in half of each experimental group according to the recommendations by the Society of Laboratory Animal Science (GV-SOLAS)^31^. Belonging to the group of NSAID, meloxicam is well known as a COX inhibitor, suppressing the prostaglandin synthesis. Meloxicam is approved in Germany for treating inflammation and pain in guinea pigs, cats, dogs, horses, cattle and pigs^58^.

#### BrdU injection

On 3–5 dpi, BrdU (Sigma (B5002)) was administered as a well-studied marker of cell proliferation (Fig. 8). It serves as a thymidine analogue with higher affinity and is incorporated into dividing cells during the S phase of the cell cycle. We tested two BrdU protocols: Animals were randomized and given either 50 mg/kg i.p. once per day in this period, or one injection of 150 mg/kg i.p. at 4 dpi. Since the results did not differ between the two groups, they are shown together. BrdU must be prepared freshly to prevent the dissolved powder from precipitating. We choose a stock solution of 5 mg/ml PBS for obtaining a suitable injection volume for i.p. injection in mice based on the national recommendation for substance application in laboratory animals GV-SOLAS^59^. BrdU was weighed and then slowly dissolved in 37 °C warm PBS.

#### Seizure monitoring

Monitoring for handling-induced seizures comprised one hour twice daily (between 9 and 12 AM and 2 and 5 PM; Fig. 8). Generalized motor seizure severity was scored using a modified Racine scale^20^ for evaluation of the development, the timing, intensity and the number of generalized acute seizures: facial movements and head nodding (score 1 + 2), myoclonic twitches or unilateral forelimb clonus (score 3), tonic–clonic convulsions (score 4), loss of righting reflexes (score 5), excessive running and jumping (score 6).

#### Monitoring of well-being, nest building, and body weight

Animals were assessed for well-being at least once daily at a constant time point in the morning according to a scoring system ranging from 0 to C, with C being the humane endpoint (Fig. 1). Evaluated parameters included: Nutritional status, body weight, exterior condition, cardiorespiratory condition, behavior and movements, digestive system, pain, and injection- or infection-associated symptoms (Supplementary Fig. 1). The overall score for the day was the highest score given for any of the above categories. If distress was present in multiple categories or lasted multiple days, the next higher score would have been applied according to the instructions in Supplementary Fig. 2, e.g. three “A” scores on the same day would lead to a score of “B”, as well as a prolonged time span of score “A” (see Supplementary Fig. 2). The respiratory and heart rate were not quantitatively measured if observation of the animal was inconspicuous. The animals with impaired well-being were observed every 8 to 12 h and never left unattended overnight for longer than 14 h. Additionally, those animals were offered soft food and eating behavior was observed to ensure food intake. The instructions for action can be traced in the supplementary file (Supplementary Fig. 2). For further analyses of well-being, nesting behavior was scored in a subgroup of mice (n = 33). The importance of nest building for heat conservation as well as for reproduction and shelter – and therefore survival—makes the nest building abilities an accurate measurement of mouse well-being. After virus injection, each mouse was single housed in a freshly equipped testing cage containing exactly 7 g of nesting material, but no additional environmental enrichment. The test used commercially available cotton swabs and tissue. Each morning from 1 to 7 dpi, nest building was assessed on a scale from 1–5 as proposed by Deacon (2006) (see Supplementary Fig. 3)^23^. Scoring was executed by three experimenters blinded to the treatment group and then averaged. On the last day of testing, the unused nesting material was weighed and compared to the used material. Nest building is a multifaceted behavior that involves intricate interactions between an animal and its environment, resulting in significant variability in nest construction. For instance, some mice may construct what appears to be a “grade 5” nest, yet still leave over 10% of the material unshredded. The usual score for nesting achieved in different healthy mouse strains is between 3.5 and 4.5^23^.

### Sample collection

At 7 dpi mice were euthanized by isoflurane inhalation. The depth of anaesthesia was assessed through breathing observation and tail pinch. In order to reliably determine the death of the mice before perfusion, we waited an additional minute after respiratory arrest. The heart continues to beat or fibrillate for several minutes after respiratory arrest. The thorax was carefully opened, and the heart mobilized. While the heart was kept in place with the help of tweezers, the pump needle was inserted at the apex of the left ventricle, pointing directly into the ventricle. Next, blood was removed with PBS for 3 min followed by fixation with 4% paraformaldehyde (PFA) for 6 min. Successful perfusion could be evaluated by the stiffness and paleness of organs. Access to the brain was attained through cutting along the foramen magnum and carefully resecting skull plates starting from the sulcus centralis. For storage, brains were preserved in 4% PFA overnight and then transferred to 30% sucrose for dehydration until further processing.

For immunolabeling of specific cells, brains were cut into 40 µm thick coronal slices using a freezing microtome (HM 400, MICROM GmbH, Germany) and divided into 8 series to represent the hippocampal dorso-ventral extent per vial. Samples were stored at − 20 °C in cryoprotective solution (30% glycerol, 30% ethylene glycol and 40% PBS (Roth, Germany)).

### Immunohistochemistry

Microglia activation as one leading actor of the innate immune response is known to be increased in viral encephalitis^19,36^. Among other things, they mediate further immune processes through the secretion of immune mediators, but they also have a neuroprotective effect on neurons damaged by seizures^57,60^. Therefore, we used Iba1, a commonly used marker for microglia, to assess if a similar activation could be reproduced under the use of meloxicam in this study. Furthermore, astrocytes are the most common class of glial cells in the central nervous system and play a major role in immune defense, such as the blood–brain barrier, but also in the uptake of neurotransmitters from the synaptic cleft. Under certain pathological conditions, such as epilepsy, astrocytes can become hypertrophic, which is known as astrogliosis. Using immunofluorescent labeling, we therefore examined the cell density and cell size of GFAP^+^ cells with regard to their changes under the influence of meloxicam. Finally, in models of temporal lobe epilepsy, it is known that the brain undergoes substantial neuronal cell loss with a focus on interneuron decline mainly in GABAergic neurons. As a consequence, excitatory signals cannot be properly inhibited, leading to a state of hyperexcitability and higher risk of seizures^61^. Consequently, we also wanted to assess neurodegeneration in the hippocampus, and used the marker NeuN to visualize adult neurons.

Brain sections were first washed three times free-floating in wash buffer (PBS Tween 0.5%) to remove cryoprotective solution. To allow the anti-BrdU antibody access to the BrdU within the DNA, samples were pre-treated with a denaturation step in HCL 2 M for 15 min at constant temperature of 37 °C^62^. After washing, the sections were incubated in blocking buffer for two hours, containing bovine serum albumin, normal goat serum, glycine and Triton-X diluted in PBS, preventing non-specific binding of antibodies.

Sections were incubated with primary antibodies at 4 °C overnight: rat anti-BrdU antibody (1:250; Abcam; ab6326) was selected for the visualization of proliferating cells. Co-labeling runs used rabbit anti-NeuN (1:1000; Thermofisher; 14H6L24) for mature neuronal cells or rabbit anti-Iba1 (1:1000) marking microglia. Secondary antibodies were incubated for 2 h: Alexa Fluor 568 anti-rat antibody (1:500; Invitrogen; Thermofisher; A-11077), Alexa Fluor 488 anti-rabbit antibody (1:1000 or 1:1500; Invitrogen; A-11008). After incubation of the secondary antibodies, a Mix-n-Stain™ CF™647 kit (Sigma-Aldrich, Germany) was used to label astrocytes (mouse anti-GFAP, chosen dilution 1:2000 and conjugated with Alexa Fluor-647). Sections were then mounted and slides were coverslipped with Fluoromount-G™ mounting medium, containing DAPI. When not otherwise stated, all incubations were done on ice to reduce background. Exclusion of primary antibody was used as negative control.

### Imaging

For image acquisition, brain sections containing the hippocampus were used. Single plane images of BrdU, Iba1, GFAP and NeuN labeling were created by using fluorescent microscopes (Leica DMi8®, Leica, Germany). For each section, the hippocampus was visualized at a magnification of 10 × and 5 × for NeuN imaging respectively. In each channel, the focus was corrected and one image including all channels was acquired either with a 1.34 × 1.34 mm or 1.22 × 0.97 mm field of view. All settings were kept constant throughout the acquisitions and different groups. Each day of acquisition involved a cross section through all experimental groups.

### Imaging analysis

Experimenters analyzing immunofluorescent images were blinded to the animal ID and experimental group. Images were analyzed in a random order with ImageJ. By setting a region of interest (ROI) around the DG utilizing the DAPI channel, the area was measured and used for quantitative assessment of cell density and average cell area for the markers of interest. Cell counting within the ROI was performed on a single plane channel on the marker of interest with the use of the “Analyze particles” plugin. Briefly, the mean fluorescence in each sample was measured and then averaged and doubled over the whole experimental data set, creating a cut-off threshold which excludes unwanted background signals. Utilizing this threshold, in a 2D image cells were automatically counted starting at a particle size equal to the smallest cells of interest represented in the channel, averaged from all images to analyze both the number and the area of marked labeling. Respectively, particle size expressed as microns was set to 7—infinity in BrdU labelled cells, 13—infinity for marked Iba1 cells and 3—infinity for the labelled GFAP^+^ cells to filter undesired background signaling. In the channel corresponding to the selected staining, images were corrected by setting the “Gamma filter” to 1.5 and in addition the “Gaussian Blur” to 2 in Iba1 analyses to improve image quality. In few samples, slightly larger numbers and bigger fluorescent areas of Iba1 positive cells resulted in fused areas involving multiple cells not identified as individual counts. To overcome this bias towards higher area and less cell count, samples were corrected each by dividing larger areas by the average cell count excluding the mentioned and adding the subsequent number of cells. Resulting cell counts were then calculated to a comparable area of 1 mm^2^ and all data was averaged over each animal for the use in statistics.

Neurodegeneration was analyzed via NeuN-labeling based on a semi-quantitative scoring system to determine potential loss of mature neurons in the CA areas of the hippocampus. From each animal, the hippocampus closest to the region according to Bregma -1.70 was selected and scored according to Bröer et al. 2016: 0 = no obvious damage; 1 = slightly abnormal appearance of the structure without clear evidence of visible neuron loss; 2 = lesions involving < 20% of neurons of the whole structure; 3 = lesions involving 20–50% of neurons; and 4 = lesions involving > 50% of neurons^19^. Analyses were performed by an experimenter blinded to animal ID and group allocation.

### Seizure analyses

Seizure analysis was performed by an experimenter blinded to the treatment twice daily during the light phase. Number and intensity of seizures were reported using a modified Racine Score from 0 to 6 (Fig. 1). If during observation periods animals did not seize spontaneously, handling was intensified as a trigger. For most analyses, the scores were added as cumulative seizure score starting at a score 3 and were compared between groups and days, because Racine stage 3 seizures can be easily identified visually^5^. Nevertheless, we also wanted to get an idea if animals with a score of 1 or 2 showed some differences in experimental readouts and included them in separate statistical analyses.

### Statistics

Before unblinding, all values were checked and, if necessary, individual data points were excluded (e.g., immunohistological labeling incorrect, injection sites damaged during processing). Per animal, 7 to 8 slices representing the hippocampal extent were analyzed and averaged. One animal was excluded from immunohistochemical analyses after viewing of acquired images due to tissue abnormalities in the lateral ventricles. All statistical analyses were performed with GraphPad Prism version 10. Data were tested for normality using the Shapiro–Wilk test and tested for variability using Brown-Forsythe test and Bartlett’s test. Multiple comparisons between normally or non-normally distributed data were further analyzed using either a one-way ANOVA followed by Tukey’s post-hoc test or Šídák's test and, if not normally distributed, a Kruskal–Wallis ANOVA followed by Dunn’s post-hoc test. If the standard deviation distribution was variable, the data were analyzed using Welch’s ANOVA test followed by a Dunnett’s test. Time-dependent analyses were performed using two-Way ANOVA or mixed-model-analysis. Seizure incidence was tested by Fisher’s exact test. Daily body weight was analyzed using repeated measures (RM) ANOVA. For comparisons between two groups, either a Student’s t-test or an unpaired Mann–Whitney test was used. P ≤ 0.05 was considered significant and the confidence interval (CI) was set to 95%. Correlation analyses were performed utilizing either the Pearson correlation coefficient or the Spearman correlation method, depending on data distribution. All data is shown as mean + /– SEM if not otherwise stated.

## Supplementary Information

Supplementary Figures.

## Data Availability

The datasets generated during and/or analyzed during the current study are available from the corresponding author on reasonable request.
