# Supplementary material for: Testing perioperative meloxicam analgesia to enhance welfare while preserving model validity in an inflammation-induced seizure model
Source: Sci Rep. 2024 Dec 19;14:30563. doi: 10.1038/s41598-024-81925-7 (PMC11659391; doi:10.1038/s41598-024-81925-7)
Supplement: Supplementary file 1 — Supplementary Figures. [file 41598_2024_81925_MOESM1_ESM.docx]

**Supplementary Figure 1: Assessed parameters for well-being.** Scored as 0-C for unaffected, mild, moderate, or severe impact on parameters such as exterior condition, behavior, weight, pain, health**.**

| **No distress (0):**   - Monitoring frequency every 12-24h. Animals kept overnight at maximum 24 hours without observation.   **Mild (A):**   - Monitoring frequency every 8-12 unless symptom-dependent intervals are shorter. Animals kept overnight at maximum 14 hours without observation/monitoring. - Interventions may be necessary (e.g., analgesia, warmth, facilitated food/water intake on the floor, circulatory stabilization (e.g., s.c. substitution of fluids), compensation for energy deficits, antibiotics) - Information to responsible person - From 3 A criteria, the instructions for B apply   **Moderate (B):**   - Monitoring frequency every 8 h unless symptom-dependent intervals are shorter. Animals kept overnight maximum 10 hours without observation/monitoring. - Interventions may be necessary (e.g., analgesia, warmth, facilitated food/water intake on the floor, circulatory stabilization (e.g., s.c. substitution of fluids), compensation for energy deficits, antibiotics, anticonvulsants) - The animal(s) shall be presented to the responsible person and/or veterinarian within 3 h. - From 3 B criteria, which individually would allow a maximum observation time of 72 h, a maximum observation time of 48 h applies and then the instructions for C. For the other criteria, 12 or 24 h observation time applies as indicated.   **Severe (C):**   - The animal must be removed immediately from the experiment and be killed. |
| --- |

**Supplementary Figure 2: Instructions for action, evaluation of well-being score.** Additional observations or interventions with fluids, antibiotics, and extra food supply might be necessary dependent on the score.

| **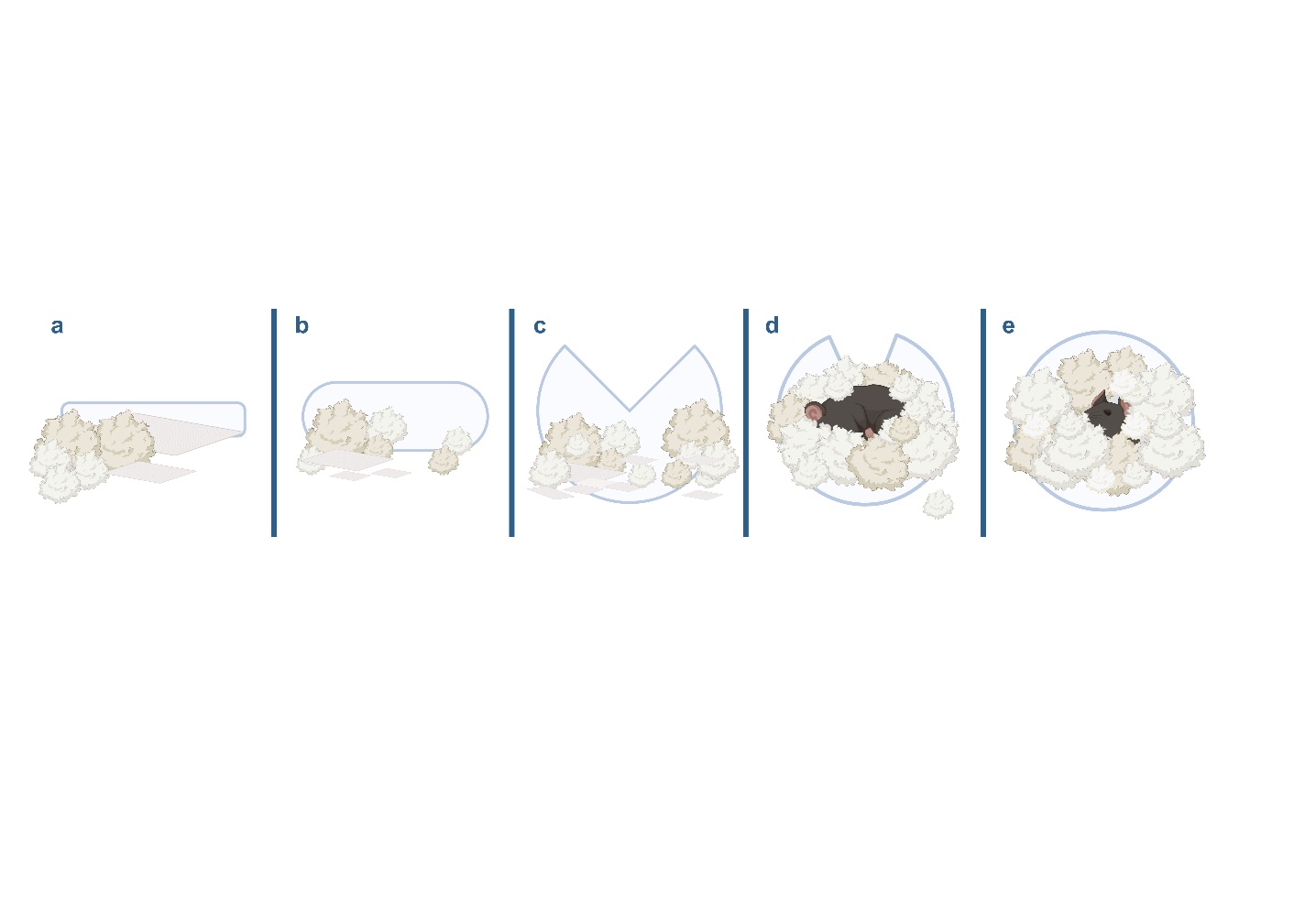** |
| --- |

**Supplementary Figure 3: Nesting score as proposed by Deacon (2006).** Once daily assessment to a fixed time point in the morning. a) Nestlet not noticeably touched (more than 90% intact). b) Nestlet partially torn (50–90% remaining intact). c) Nestlet mostly shredded but often no identifiable nest site: less than 50% of the Nestlet remains intact, but less than 90% is within a quarter of the cage floor area. The material may sometimes be in a broadly defined nest area. d) An identifiable but flat nest: more than 90% of the Nestlet is torn and the material is gathered into a nest within a quarter of the cage floor area, but the nest is flat, with walls higher than mouse body height (of a mouse curled up on its side). e) A (near) perfect nest: more than 90% of the Nestlet is torn and the nest is a crater, with walls higher than mouse body height or above.
